# Conductive Nerve Guidance Conduits Loaded With Adipose Mesenchymal Stem Cells for Peripheral Nerve Regeneration

**DOI:** 10.1002/smmd.70025

**Published:** 2025-12-10

**Authors:** Hong Cheng, Yangnan Hu, Menghui Liao, Xinyi Pang, Hui Zhang, Meihan Yu, Bin Zhang, Yu Wang, Huan Wang, Tingting Liu, Renjie Chai

**Affiliations:** ^1^ Department of Otolaryngology Head and Neck Surgery, Zhongda Hospital, State Key Laboratory of Digital Medical Engineering, Jiangsu Provincial Key Laboratory of Critical Care Medicine School of Public Health School of Medicine Advanced Institute for Life and Health Southeast University Nanjing China; ^2^ Co‐Innovation Center of Neuroregeneration Nantong University Nantong China; ^3^ School of Medical Engineering Affiliated Zhuhai People's Hospital Beijing Institute of Technology Zhuhai China; ^4^ College of Food Science and Engineering Nanjing University of Finance and Economics Nanjing China; ^5^ Department of Otolaryngology Head and Neck Surgery Nanjing Drum Tower Hospital Clinical Medical College of Traditional Chinese and Western Medicine Nanjing University of Chinese Medicine Nanjing China; ^6^ Wenzhou Institute University of Chinese Academy of Sciences Wenzhou Zhejiang China; ^7^ The Eighth Affiliated Hospital Sun Yat‐sen University Shenzhen China; ^8^ Department of Neurology Aerospace Center Hospital School of Life Science Beijing Institute of Technology Beijing China; ^9^ Institute for Stem Cell and Regeneration Chinese Academy of Sciences Beijing China; ^10^ Southeast University Shenzhen Research Institute Shenzhen China

**Keywords:** adipose‐derived stem cells, hydrogel, multi‐walled carbon nanotubes, nerve guidance conduits, nerve regeneration

## Abstract

Peripheral nerve injury (PNI) presents a significant clinical challenge due to the intrinsic limitations of nerve regeneration and poor functional recovery. Although nerve guidance conduits (NGCs) offer a promising alternative to autografts, their therapeutic efficacy is often constrained by insufficient bioactivity and electrical conductivity. To address these dual deficiencies, we engineered an electroactive living nerve conduit by integrating silk sericin (SS)‐modified carbon nanotubes (SCNTs) with adipose‐derived stem cells (ADSCs). The SCNTs serve as a conductive scaffold, whereas the ADSCs provide a sustained release of neurotrophic factors. This design creates a synergistic microenvironment to promote neuronal maturation and axonal regeneration. In an experimental rat model featuring a 10‐mm sciatic nerve gap, ADSC/SCNT/RAM NGCs demonstrated regenerative performance comparable to autografts, facilitating axon connection and recovery of motor functions. Histological assessment revealed that the implanted ADSC/SCNT/RAM NGCs promoted the most extensive nerve and axon regeneration among all groups, as evidenced by the significantly higher counts of S100 calcium‐binding protein B (S100‐β)‐positive cells (10,152 ± 986.00) and Neurofilament Protein 200 (NF200)‐positive cells (11,517 ± 795.70). Corroborating these histological findings, functional analysis demonstrated that the ADSC/SCNT/RAM group achieved the highest sciatic nerve function index (SFI) at 12 weeks post‐surgery (−58.06 ± 1.46), a value comparable to the Autograft group (−57.73 ± 1.80). This strategy proposes a promising tissue‐engineered alternative to autografts for nerve repair.

## Key Points

- The combination of hollow NGCs and SS‐modified MCNTs provides excellent mechanical and electrical properties of NGCs.
- Introducing ADSCs into the inner wall of conductive NGCs secretes abundant neurotrophic factors that synergistically promote neuronal maturation.
- Electroactive living NGCs loaded with ADSCs and SCNTs markedly enhanced peripheral nerve regeneration and restored axonal connectivity and motor function to a level comparable to the Autograft group.

## Graphical abstract

An electroactive living nerve guidance conduit integrating conductive SCNTs with neurotrophic ADSCs was engineered. This design achieved nerve regeneration and functional recovery comparable to autografts in a rat sciatic nerve gap model.

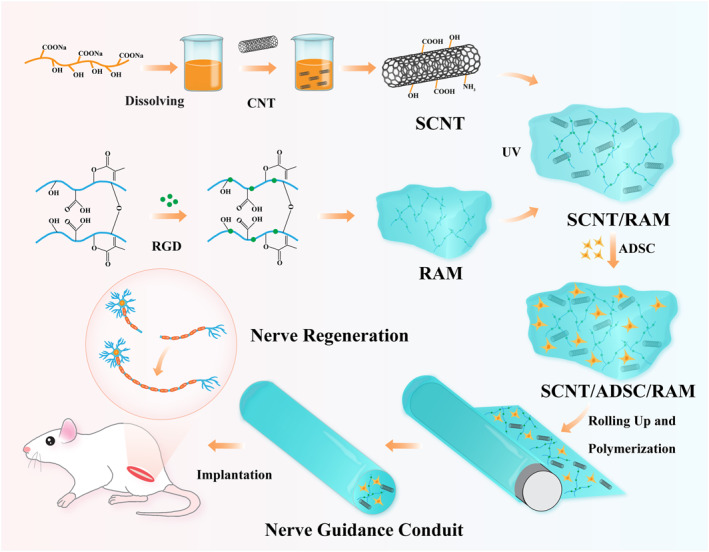

## 1 Introduction

Peripheral nerve injury (PNI) is a critical clinical dilemma, with nerve defects inducing sensory and motor dysfunction, placing a heavy financial strain on public health resources [1, 2, 3]. The primary clinical intervention for PNI currently is surgical treatment, specifically end‐to‐end nerve sutures. However, surgical suturing has inherent limitations, such as misplaced axonal connections, nerve curls, and excessive tension caused by nerve suturing [4, 5], which leads to unsatisfactory clinical outcomes. Conversely, tissue‐engineered nerve guidance conduits (NGCs) represent a burgeoning field of research, offering a highly promising strategy to facilitate regeneration and functional recovery following PNI [6, 7, 8]. This potential is attributed to their ability to integrate both biochemical and physical cues, which collectively facilitate the establishment of a supportive microenvironment for axonal regeneration [9, 10]. Notably, the inclusion of bioactive molecules, including neurotrophic factors, has proven effective in promoting nerve regeneration [11, 12]. Nevertheless, existing methods of constructing NGCs to facilitate nerve regeneration still face several problems [13, 14]. On the one hand, the release of bioactive factors encapsulated in hydrogels is time‐sensitive, while the growth of peripheral nerve tends to be slow, resulting in insufficient and unsustained support for nerve growth [15]. On the other hand, complex electrical signals are integral to the regulation of nerve growth and regeneration processes [16, 17]. These signals, capable of being transmitted or generated under external stimulation to activate key intracellular signaling pathways, constitute a critical regulatory mechanism [18]. Indeed, recent studies have demonstrated that conductive materials, such as carbon nanotubes (CNTs) and graphene, can effectively stimulate neural‐derived cells, including the differentiation of PC12 cells and the proliferation of Schwann cells (SCs) [8, 19]. However, a significant challenge lies in the poor dispersibility of these conductive materials within hydrogel matrices, which adversely diminishes their electrical conductivity. Consequently, the structural tunability and superior processability of conductive polymers are of paramount importance for the fabrication of conductive NGCs. Nevertheless, a critical limitation persists in the majority of current NGC designs: the lack of sustained release of bioactive factors is often coupled with an absence of regulatable electroactive functionality. Consequently, novel NGCs necessitate development to address these limitations.

Herein, we propose an electrically conductive living NGC incorporated with multi‐walled carbon nanotubes (MCNTs) and stem cells to enhance nerve regeneration. MCNTs exhibit high aspect ratio structures and excellent electrical conductivity, making them widely used in the fabrication of bio‐conductive materials to improve the mechanical and electrical behaviors of hydrogels [20, 21]. Moreover, MCNTs have shown the capability to enhance the differentiation of PC12 cells and stem cells into neurons by facilitating the transmission of electrical signals and enhancing neuronal excitability [22]. However, MCNTs are typically hydrophobic and not easily dispersed in hydrogels and other aqueous solutions, adversely impacting the homogeneity of hydrogels. The carboxyl (–COOH) and hydroxyl (–OH) groups present in silk sericin (SS), a type of silk cocoon protein, can non‐covalently graft onto the amino group (–NH_2_) of CNTs, thereby improving the dispersion of MCNTs [23]. In addition, the application of adipose‐derived stem cells (ADSCs), acquired from adipose tissue, constitutes a significant therapeutic strategy within neuroregenerative processes in the peripheral nervous system [24, 25, 26, 27, 28]. ADSCs exhibit the ability to secrete abundant neurotrophic factors, such as nerve growth factor (NGF), brain‐derived neurotrophic factor (BDNF), and glial cell line‐derived neurotrophic factor (GDNF), which are recognized for their role in supporting neuronal growth, proliferation, and migration of SCs during PNI repair [29, 30, 31, 32]. Therefore, we hypothesize that integrating ADSCs with SS‐modified CNTs (SCNTs) within an NGC will create a synergistic system that simultaneously provides sustained neurotrophic support and enhanced electroactive functionality, addressing the key limitations of current conduit designs.

**SCHEME 1:**
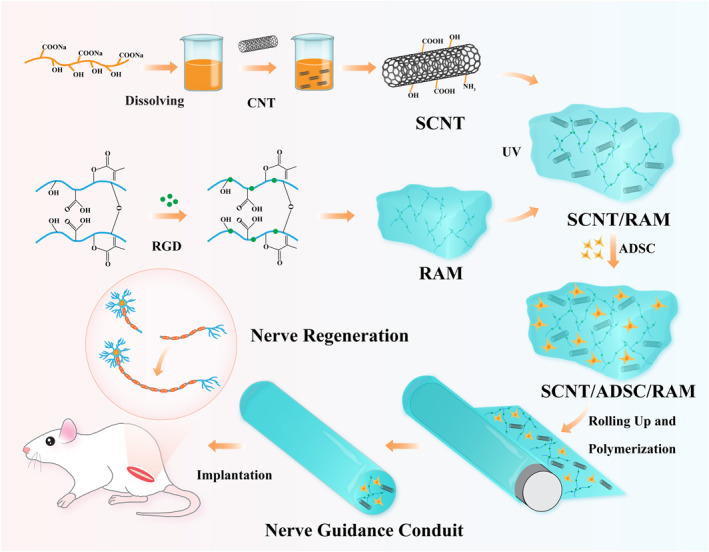
Schematic diagram of a conductive NGC loaded with ADSCs for peripheral nerve regeneration.

In this work, we developed bioactive NGCs integrating ADSC, SCNT, and RGD‐modified methacrylate‐acylated sodium alginate (ADSC/SCNT/RAM) with high electrical conductivity, favorable mechanical properties, and good biocompatibility to support the regeneration and functional reconstruction in a rat model of PNI as shown in Scheme 1. Initially, SCNTs were incorporated into RAM hydrogel films by hydrogen‐bond cross‐linking. Subsequently, ADSCs were grafted onto the surface of the films, and hollow NGCs were prepared by simply rolling the film. The resulting NGCs inherited the high electrical conductivity of MCNTs, which promoted neuronal maturation. Furthermore, NGCs loaded with ADSCs demonstrated sustained release of neurotrophic factors, improving nerve nourishment and axonal regeneration. Our results revealed that ADSC/SCNT/RAM NGCs effectively facilitate the proliferation, migration, and differentiation of SCs and PC12 cells. Additionally, by establishing a sciatic nerve injury model, it was demonstrated that the prepared ADSC/SCNT/RAM NGCs could foster the bridging of the damaged nerves and the restitution of motor functions in animals with high efficacy. These features suggest that ADSC/SCNT/RAM NGCs hold significant potential for fostering axonal regeneration and functional reconstruction in PNI, underscoring their value for applications in biological tissue engineering.

## 2 Methods

### 2.1 Reagents and Animals

Male Sprague Dawley (SD) rats, weighting 200 g, were acquired through Qinglongshan Animal Co. Ltd (Nanjing, China). A 2% (w/w) sodium alginate solution was obtained from Sigma (USA). DMEM medium was acquired from GibcoBRL (Gaithersburg, MD, USA). Fetal bovine serum (FBS) was obtained from Senbeijia (China). The GDNF, BDNF, and NGF Enzyme‐linked immunosorbent assay (ELISA) kits were obtained from Mlbio (Shanghai, China). The NGF was procured from PeproTech (USA). 0.25 wt% trypsin was acquired from GibcoBRL (Gaithersburg, MD, USA). The Cell Counting Kit‐8 (CCK‐8) and the LIVE/DEAD Viability/Cytotoxicity Kit were obtained from Beyotime (China). Mouse anti‐βIII‐tubulin, neuroglia marker S100 calcium‐binding protein B (S100‐β), and rabbit anti‐Neurofilament Protein 200 (NF200) were sourced from Abcam (UK).

### 2.2 Preparation and Characterization of RGD‐AlgMA Hydrogel

To achieve a 1% (w/v) solution of very low‐viscosity sodium alginate, 2 g of the polymer was dissolved in 200 mL of deionized water maintained at 50°C. Continuous stirring was applied at 400 rpm throughout the dissolution process until the polymer was fully dissolved. Subsequently, 32 mL of methacrylic anhydride (MA) was added to the reaction mixture in a dropwise fashion. Throughout the 48‐h reaction period, which was conducted at 4°C, the pH of the mixture was maintained at 8 by the continuous addition of 5 M NaOH. Upon completion of the reaction, the solution was dialyzed against deionized water for 6 days to remove low‐molecular‐weight species utilizing a membrane with a 13 kDa molecular weight cut‐off, with the dialysate replaced twice daily. Post‐dialysis, the solution was lyophilized to yield a white, soft AlgMA solid. To prepare the RGD‐AlgMA precursor solution, AlgMA was formulated at a 6% concentration in the presence of the photoinitiator LAP. Subsequently, the AlgMA solution was combined with Pep‐RGDfKAC at a molar ratio of 4:1. This precursor was subsequently photo‐solidified into a hydrogel through blue light irradiation for 30 s. Nuclear magnetic resonance hydrogen spectroscopy (^1^H NMR) was implemented to analyze the chemical modification of MA on alginate at a resonance frequency of 400 MHz. Scanning electron microscopy (SEM) was applied to investigate the apparent morphology of the hydrogels.

### 2.3 Preparation and Characterization of SCNT

SS was solubilized in deionized water to yield a 2% solution, to which CNTs were subsequently added and ultrasonicated for 30 min at a power of 100 W. Following thorough mixing, SCNTs with high dispersibility were obtained. Morphological changes resulting from the modification of CNTs with SS were detected by transmission electron microscopy (TEM). The elemental distribution of SCNTs was analyzed through Energy Dispersive X‐Ray Spectroscopy (EDX) elemental mapping. The successful modification of CNTs by SS was confirmed using X‐ray diffraction (XRD) and Fourier‐transform infrared spectroscopy (FTIR). The electrical conductivity of SCNTs at different concentrations was measured using a four‐probe resistance tester.

### 2.4 Construction and Characterization of ADSC/SCNT/RAM NGCs

NGCs were prepared by rolling an ADSC‐loaded SCNT/RAM hydrogel around a silicone tube template. Briefly, ADSC cells were cultured on the interface of SCNT/RAM hydrogel, and after 2 days, the hydrogel was wrapped around a silicone tube template of a specific size and rolled along the silicone tube to prepare NGCs, with the side containing the cells serving as the inner wall of the conduit. A small amount of RAM solution was applied to the seams, and the seams of the conduit were bonded using blue light irradiation.

The microstructure of the prepared ADSC/SCNT/RAM NGCs was observed using SEM. Characterization of the dynamic swelling behavior commenced after vacuum freeze‐drying the composite hydrogel for 24 h to ascertain its initial dry weight (W0), which was then placed in PBS at 37°C, and the wet weight (WS) was recorded periodically, during which the liquid remaining on the surface of the hydrogel was removed. The swelling rate of the composite hydrogel was calculated as: $\text{Swelling}\;\text{ratio}=\frac{\text{WS}}{W0}\times 100\%$. For the assessment of the degradation behavior of the composite hydrogel, the W0 was recorded before immersion in 1 KU/mL of collagenase, allowing it to degrade at 37°C. The collagenase solution was then filtered out at the appropriate time point, and the hydrogel was rinsed with deionized water, vacuum freeze‐dried, and weighed (Wd). The degradation rate of the composite hydrogel was calculated using the formula: $\text{Degradatio}n\;\text{rate}\frac{W0-\text{Wd}}{W0}\times 100\%$.

For the implantation of cells within the ADSC/SCNT/RAM NGCs, a multi‐step fabrication process was employed. Initially, a hydrogel film was fabricated and subsequently surface‐functionalized with laminin to promote robust cell adhesion. Following a 2‐day cell culture period on this functionalized film, the membrane was rolled around a silicone tube serving as a temporary mandrel to form a tubular conduit. To mitigate potential cell loss or damage during this rolling process, a secondary cell seeding strategy was implemented. Specifically, after the NGC was formed, a suspension of additional cells was injected into the lumen to ensure uniform and sufficient cellularization.

### 2.5 Cell Culture

PC12 cell culture: PC12 cells were cultivated in RPMI 1640 medium supplemented with 10% FBS and 1% penicillin/streptomycin at 37°C with 5% CO_2_. Passaging was performed every 2 days.

ADSCs’ culture: The inguinal fat pads of 2‐3‐week‐old SD suckling mice were extracted under aseptic conditions and digested using 0.1% collagenase type I for 40–60 min in a water bath shaking at 37°C until the solution achieved a milky consistency. The cell suspension underwent initial centrifugation at 1500 rpm for 3 min. Following this, the liquid phase was discarded by aspiration, and the cell pellet was re‐suspended and diluted in fresh culture medium. A subsequent centrifugation was carried out at 1500 rpm for 3 min. In standard culture protocols, medium changes were conducted every 3 days to eliminate unattached cells, while cell passaging occurred once 80% confluence was achieved.

RSC96 cells culture: For routine culture, RSC96 cells were cultivated in high‐glucose DMEM supplemented with 10% FBS and 1% penicillin/streptomycin, with passage performed every 3 days.

### 2.6 Live/Dead Staining and CCK‐8 Assay

Cell viability following 3‐day co‐culture with the different material groups was investigated employing the LIVE/DEAD Cell Viability/Toxicity Assay Kit. The supernatant was excluded, and the cells were rinsed with PBS to remove non‐adherent cells and dead cells. Subsequently, the cells were incubated with Calcein‐AM and Propidium Iodide (PI) for 30 min, and cell viability was then assessed under a fluorescence microscope. In addition, cell viability was quantitatively evaluated using the CCK‐8 assay. The cells were incubated with CCK‐8 reagent for 2 h, and cell viability was quantified by recording the absorbance at 450 nm.

### 2.7 EdU Test

The effects of mitomycin and ADSC at varying concentrations on RSC96 cells proliferation were determined using an EdU Imaging kit. Briefly, EdU incorporation was assessed by incubating RSC96 cells with diluted EdU reagent for 4 h. This was followed by a 45‐min reaction with detection buffer at 20°C–25°C, as per the manufacturer's protocol. Proliferating cells were visualized under a confocal microscope.

### 2.8 Differentiation of PC12 Cells

PC12 cells differentiation was evaluated on tissue culture polystyrene (TCPs), RAM, SCNT/RAM, and ADSC/SCNT/RAM substrates. Cells were seeded and allowed to proliferate for 24 h, and then subjected to 100 ng/mL of NGF to induce differentiation. Five days post‐treatment, differentiated cells were fixed and analyzed by Tuj‐1 immunofluorescence staining.

### 2.9 Migration of RSC96 Cells

The migration capacity of RSC96 cells under ADSC influence was assessed using the Transwell system. Briefly, ADSCs were inoculated into the upper transwell compartment, while RSC96 cells were inoculated on coated slides in 24‐well plates. The migration of RSC96 cells was observed at specific time points.

### 2.10 Cell Immunofluorescence Analysis

Following fixation with 4% paraformaldehyde for 40 min at 20°C–25°C, cells underwent 2 washes with PBST. Subsequently, cells were permeabilized and blocked for 30 min at 37°C in a blocking solution. Primary antibodies, specific to the targets of interest, were incubated with the cells overnight at 4°C. The cells were subsequently incubated with the secondary antibody for 2 h at 37°C. PBST washes (3 min each, 3 times) were performed between all sequential steps.

### 2.11 Sciatic Nerve Amputation and NGC Implantation Surgery

To analyze the nerve regeneration effectiveness of the processed NGC, a sciatic nerve injury model with a defect of 10 mm in length was constructed in adult SD rats weighing 200 g. All surgical procedures were performed under aseptic conditions. The rats were anesthetized and maintained with 3% and 1.5–2% isoflurane. Following the induction of isoflurane, the left hind limb was shaved and disinfected. A skin incision was made to expose the thigh muscle, which was gently retracted to locate the sciatic nerve. A 10‐mm portion of the sciatic nerve was carefully resected to create the defect model. The prepared NGC was sutured to both nerve stumps using 8‐0 absorbable sutures. Rats with nerve defects were stochastically distributed into 4 experimental groups: autograft group, RAM catheter treatment group, SCNT/RAM catheter treatment group and ADSC/SCNT/RAM catheter treatment group. The severed nerve endings as well as the muscle and skin layers of the Autograft group were immediately reattached with absorbable sutures. The muscle layer and skin were closed with sutures. To ensure postoperative analgesia, carprofen (5 mg/kg) was administered subcutaneously for 48 h post‐surgery. Motor function recovery was assessed by footprint analysis 12 weeks post‐surgery.

Predefined humane endpoints were established as exclusion criteria to safeguard animal welfare. Any animal exhibiting one of the following conditions was excluded from the study: (1) severe autotomy (self‐mutilation) of the denervated limb, (2) loss of > 20% of pre‐surgical body weight, (3) signs of severe systemic infection unresponsive to care, or (4) inability to access food or water. No animals met these criteria and were excluded from this study.

### 2.12 Motor Function Recovery Assessment and Trail Analysis

After twelve weeks NGC implantation, functional recovery was evaluated in all animals via walking track analysis. The CatWalk system was used to record and analyze the dynamic gait parameters at 12 weeks postoperatively. The SFI is a quantitative measure ranging from 0 to −100, where a score of 0 reflects intact nerve function and −100 signifies complete functional impairment.

### 2.13 Tissue Processing and Immunohistochemistry

Twelve weeks post‐peripheral nerve repair, rats were humanely euthanized via 2% sodium pentobarbital overdose. Regenerated sciatic nerve and bilateral gastrocnemius muscles were isolated, fixed in 4% PFA overnight, dehydrated, OCT embedded, and snap‐frozen. Seven‐micrometer‐thick tissue sections were incubated overnight at 4°C with primary antibodies specific to NF200 and S100‐β. Post‐wash with PBST, incubation with species‐matched secondary antibodies was performed at 37°C for 1 h, and cell nuclei were next labeled with DAPI. Images were acquired by laser confocal microscopy and analyzed with ImageJ. In addition, following hematoxylin and eosin (H&E) staining of gastrocnemius muscle sections, imaging was performed using a light microscope.

### 2.14 Statistical Analysis

Images were analyzed using ImageJ, and to analyze the data, visualization was carried out employing GraphPad Prism 9.0. For statistical evaluation, intergroup comparisons between two groups were made using Student's *t*‐test, whereas comparisons across multiple groups relative to a control were conducted using one‐way ANOVA followed by Dunnett's post hoc test. Statistical significance was defined as *p* < 0.05.

## 3 Results

### 3.1 Preparing and Characterizing SCNT

**FIGURE 1:**
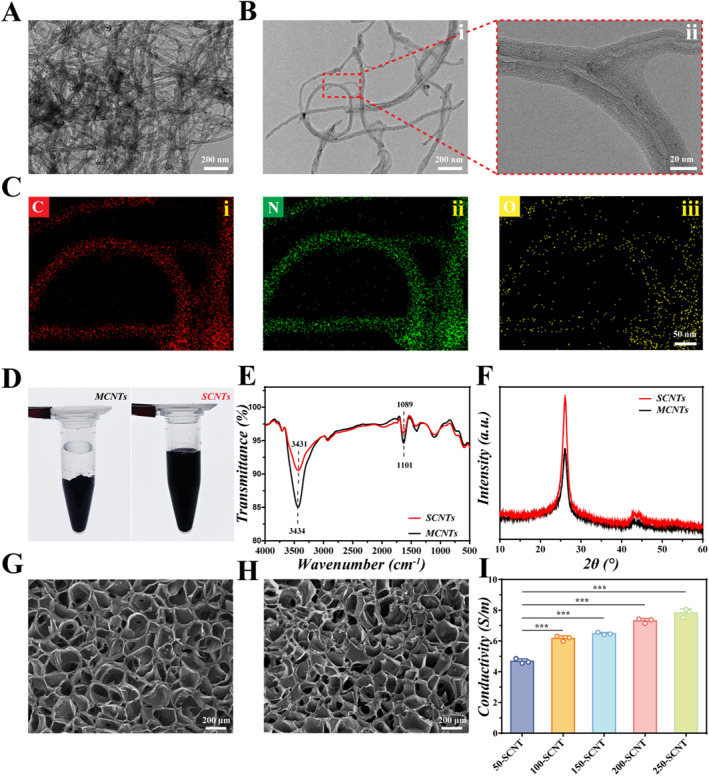
Preparation and characterization of SCNT. (A, B) TEM images of MCNTs and SCNTs, (i, ii) TEM images of SCNTs at low and high magnifications, respectively. Scale bar: 200 nm (i), 20 nm (ii); (C) EDX elemental mapping images of SCNTs, (i, ii, iii) represent the elemental analysis images for C, N, and O, respectively. Scale bar: 50 nm; (D) Bright‐field photographs depicting MCNTs and SCNTs dispersed in water; (E) FTIR spectra of MCNTs and SCNTs; (F) XRD spectra of MCNTs and SCNTs; (G, H) SEM images of RAM and SCNT/RAM; Scale bar: 200 μm; (I) Electrical conductivity of SCNT/RAM hydrogels with different SCNT contents.

The inherent hydrophobic nature of MCNTs often poses challenges in achieving uniform dispersion within hydrogels [33]. To address this issue, mixing SS powder with MCNTs serves to functionalize and modify MCNTs, and the resulting SCNTs usually have enhanced dispersibility. Morphological changes in MCNTs pre‐ and post‐SS modification were analyzed using TEM. It was revealed that unmodified MCNTs exhibited substantial aggregation due to their high aspect ratio and low hydrophilicity (Figure 1A). In contrast, the SCNTs displayed a homogeneous distribution with minimal entanglement, achieved through the adsorption of SS onto MCNT surfaces via hydrogen‐bonding interactions (Figure 1B). Meanwhile, the outcomes of elemental analysis from EDX showed a homogeneous distribution of C, N, and O elements in SCNTs, indicating the successful modification of SS on the MCNTs (Figure 1C). Figure 1D also shows that SCNTs are uniformly distributed in ddH_2_O, whereas MCNTs exhibit significant aggregation. These findings indicate the effective noncovalent modification of SS on the surface of MCNTs, thereby reducing aggregation and enhancing dispersion.

The functionalized modification of MCNTs by SS was further investigated by FTIR and XRD. The FTIR outcomes revealed hydroxyl (O‐H) stretching vibrations at 3434 cm^−1^ for the MCNTs, while the O‐H stretching vibrations of SCNTs were notably enhanced at 3431 cm^−1^ (Figure 1E). These results suggested that the increased presence of O‐H indicated the formation of hydrogen bonding between SS and MCNTs. Additionally, both the MCNTs and SCNTs exhibited carbon‐oxygen bond (C‐O) stretching vibrations at 1101 and 1089 cm^−1^, respectively. However, SCNTs displayed a higher peak intensity, implying an increase in C‐O bonds, suggesting a successful coverage of the abundant carbon‐oxygen groups present in SS onto the MCNTs. Meanwhile, the XRD patterns exhibited strong and weak diffraction peaks at 2*θ* = 26.1° and 43.1° for the MCNTs, corresponding to the (002) and (100) crystal planes, respectively (Figure 1F). Notably, the diffraction peak of SCNTs at 2*θ* = 26.1° appeared sharper, indicating an increased number of intermolecular hydrogen bonds, suggesting the successful modification of MCNTs by SS. In addition, light‐polymerizable RAM hydrogel scaffolds were synthesized by grafting MA and acrylated RGD peptides onto the sodium alginate. The chemical structure of AlgMA was elucidated through proton ^1^H NMR (Supporting Information S1: Figure S1). The SEM images of RAM hydrogels and SCNT/RAM hydrogels in Figure 1G,H showed that the pore size of the hydrogels became irregular and more closed pores appeared with the addition of SCNTs. This was attributed to the hydrogen bonding interactions between the –OH and carboxyl (COO–) groups in the RAM hydrogel and the amino, carboxyl, and hydroxyl groups in the SCNTs, which result in a higher cross‐linking density within the hydrogel. In addition, the electrical conductivity of SCNT/RAM hydrogels with varying SCNT concentrations was assessed. Figure 1I shows that the electrical conductivity of the hydrogels showed a progressive rise with the augmentation of SCNT content.

### 3.2 Preparing and Characterizing SCNT/RAM NGC

**FIGURE 2:**
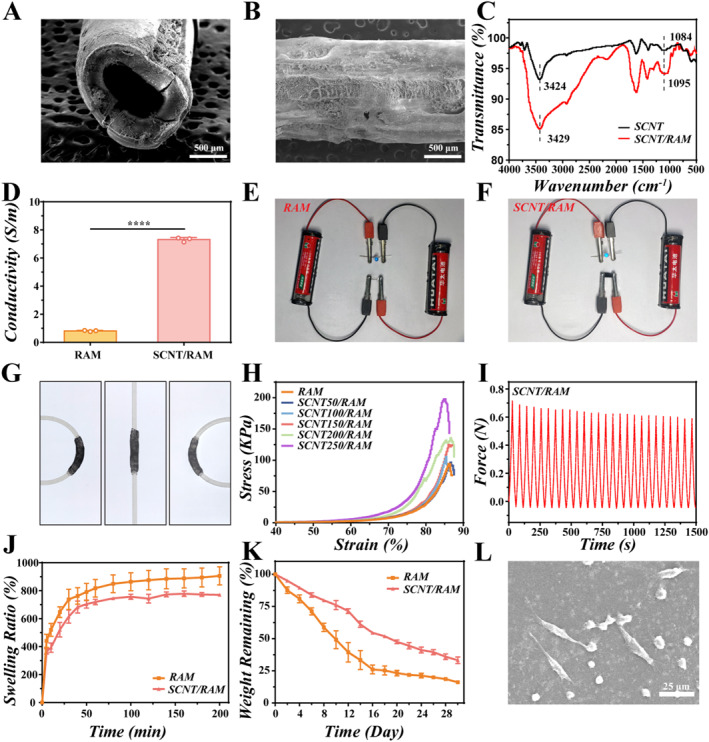
Preparation and characterization of SCNT/RAM NGC. (A, B) SEM images of prepared NGCs in longitudinal (A) and transverse (B) orientations. Scale bar: 500 μm. (C) FTIR spectra of SCNT and SCNT/RAM. (D) Electrical conductivity of RAM and SCNT/RAM NGCs; ***p < 0.001. (E, F) LED sensing clamp experiments with SCNT and SCNT/RAM hydrogels. (G) Photographs of NGCs bending in different orientations. (H) Stress‐strain curves of SCNT/RAM hydrogels at different SCNT concentrations. (I) Cyclic fatigue test results of NGCs subjected to compression cycles. (J) Swelling curves of SCNT and SCNT/RAM hydrogels. (K) Degradation curves of SCNT and SCNT/RAM hydrogels. (L) SEM image of ADSC grown on SCNT/RAM hydrogel. Scale bar: 25 μm.

The preparation of the SCNT/RAM involved rolling and curling the material around a silicone tube, which served as a template to form the desired structure. Subsequently, the RAM was used to secure the seams at the edges of the NGC, and the hollow NGC was obtained by removing the silicone tube. The morphology of the ADSC/SCNT/RAM NGC was characterized using SEM, revealing a hollow tubular structure (Figure 2A,B). The results of the FTIR spectra in Figure 2C show the stretching vibration of O‐H at 3424 and 3429 cm^−1^ and the stretching vibration of C‐O at 1084 and 1095 cm^−1^ for SCNT and SCNT/RAM, respectively, which indicate that the SCNT was successfully doped into the RAM. The electrical conductivity of the NGC was increased from 0.82 ± 0.023 Sm^−1^ to 7.31 ± 0.086 Sm^−1^ as SCNTs were doped into the RAM (Figure 2D). Light‐emitting diodes (LEDs) were utilized in subsequent experiments to evaluate the electrical current delivery performance of the SCNT/RAM hydrogel. The luminous intensity of the LED within the SCNT/RAM group was observed to substantially exceed that measured in the RAM group when the hydrogel was connected to the LED sensing clamp (Figure 2E,F). These findings verified that the incorporation of SCNT significantly boosted the conductivity of the hydrogel. The increase in conductivity can enhance the transmission of electrical signals within biological tissues, thereby promoting the healing of neural tissues. Meanwhile, with the doping of SCNTs, SCNT/RAM NGC demonstrated the ability to bend at a large angle without structural damage (Figure 2G). A range of SCNT levels (0, 50, 100, 150, 200, and 250 μg/mL) were incorporated into RAM hydrogels, which were then prepared to assess the impact of SCNT content on the hydrogels' mechanical properties. The stress‐strain curves in Figure 2H demonstrated that the compressive stress of SCNT/RAM increased with higher SCNT concentrations. Specifically, an increase in SCNT content consistently increased the compressive strength, with the SCNT 250/RAM exhibiting the highest fracture strength of about 200 kPa. Furthermore, the ADSC/SCNT/RAM NGC maintained superior structural integrity during the subsequent tests involving 25 compression cycles (Figure 2I). This indicated that the NGC possessed good mechanical durability and stability, making it a suitable candidate for implantation as a substitute for autologous nerve grafts.

In addition, the swelling and degradation characteristics of hydrogels are vital physical properties that significantly impact the outcomes of implantation procedures [34]. Figure 2J shows the swelling ratio trend of different hydrogels over time. It was indicated that both RAM and SCNT/RAM hydrogels exhibited rapid increases in swelling within 1 hour, reaching 905.80% and 770.44%, respectively. Subsequently, the swelling trend gradually decreased after 1 h until it reached equilibrium. The reduced swelling rate observed in SCNT/RAM hydrogels may be attributed to their denser structure with more restricted pores, facilitating slower water penetration. Figure 2K presents the degradation kinetics data obtained for the various hydrogels under investigation, revealing that RAM reached a maximum degradation rate of 83.87% at day 30, while SCNT/RAM exhibited a lower rate of 66.82%. This suggests the embedding of SCNT augmented the stability of the hydrogels. In addition, the SEM image illustrated ADSCs could grow in the inner wall of the SCNT/RAM NGC (Figure 2L), which provided the essential neurotrophic factors for promoting axonal regeneration.

To evaluate the biocompatibility of SCNT/RAM, the cytotoxicity of SCNT/RAM with different SCNT concentrations was assessed using a Live/Dead assay. RSC96 cells were seeded onto SCNT/RAM substrates with different SCNT contents and cultured for 48 h. Live/Dead staining subsequently confirmed significant cytotoxicity at 250 μg/mL SCNT (Supporting Information S1: Figures S2 and S3). The cytotoxicity of the prepared SCNT/RAM hydrogels was further evaluated by seeding ADSC and PC12 cells on hydrogel substrates. Comparable to TCP, no significant cell death was observed in ADSC and PC12 cells cultured on RAM and SCNT/RAM substrates, indicating good biocompatibility (Supporting Information S1: Figure S4A). Additionally, CCK‐8 experiments further confirmed the nontoxicity of SCNT/RAM, with both ADSC and PC12 cells exhibiting normal viability on RAM and SCNT/RAM substrates throughout the 48‐h culture period (Supporting Information S1: Figure S4B,C).

### 3.3 Effect of ADSC on Proliferation and Migration of RSC96 Cells

SCs are essential for the regeneration of impaired peripheral nerves, and the regeneration process largely depends on their proliferation and migrations [35, 36, 37, 38, 39]. Following axonal injury, SCs are activated and migrate to the damaged site. Driven by neurotrophic factors, they rapidly proliferate and form Bands of Büngner, which serve as a physical scaffold to guide axonal sprouting and regeneration. Furthermore, increasing evidence suggests that stem cell transplantation significantly enhances repair processes following peripheral nerve injuries [40, 41]. This effect is attributed to the paracrine potential of transplanted stem cells, which release a diverse array of neurotrophic factors. These factors subsequently promote the proliferation and migration of SCs via intercellular signaling mechanisms. ADSCs are extensively utilized in PNI repair owing to their high availability and potential to secrete neurotrophic factors. ADSCs isolated from the groin of SD suckling mice were characterized immunophenotypically. Immunofluorescence results showed that CD73 and CD105, two markers of stem cells, were highly expressed in ADSCs, while CD45, a hematopoietic marker, exhibited negative expression, suggesting the successful isolation of ADSCs (Supporting Information S1: Figure S5).

**FIGURE 3:**
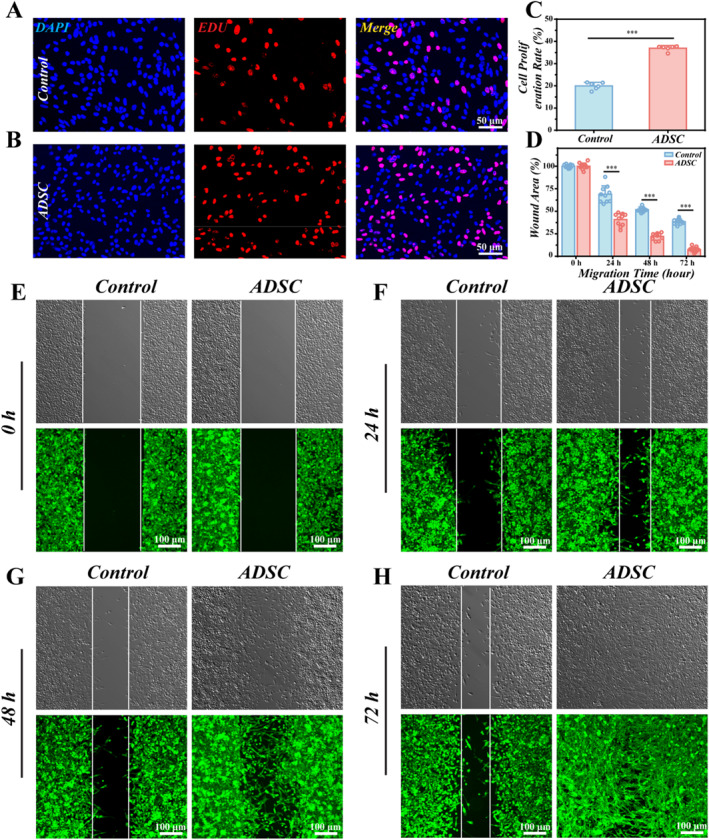
ADSC promotes the proliferation and migration of RSC96 cells. (A, B) Fluorescence images of EdU assay of RSC96 cells cultured individually (A) and co‐cultured with ADSC using the Transwell system (B). EdU (red) labels the nuclei of dividing cells, and DAPI (blue) labels the nuclei of all cells. Scale bar: 50 μm. (C) Statistics of the proliferation rate of RSC96 cells. (D) Quantitative results from the scratch assay for RSC96 cells. (E–H) Representative images of the scratch assay for RSC96 cells at 0 (E), 24 (F), 48 (G), and 72 (H) h under different culture conditions. Scale bar: 100 μm.

To evaluate the effect of ADSCs on Schwann cell proliferation, RSC96 cells were co‐cultured with ADSCs in a transwell system for 48 h and then analyzed with the EdU assay. It was observed that the proliferation rate of RSC96 cells co‐cultured with ADSCs (36.96 ± 0.49%) was higher than that of RSC96 cells cultured on TCP (19.99 ± 0.66%), which may be attributed to the neurotrophic factors secreted by ADSCs promoting the proliferation of RSC96 cells (Figure 3A–C). To further validate the proliferative effect, RSC96 cells were co‐cultured with the SCNT/RAM and ADSC/SCNT/RAM constructs. Quantitative analysis showed a significantly greater number of S100‐β‐positive cells in the ADSC/SCNT/RAM group (253.3 ± 0.88) than in the SCNT/RAM group (223.7 ± 6.01) and the TCP control (191.0 ± 6.81). This finding further corroborates the crucial role of ADSC‐derived neurotrophic factors in stimulating the proliferation of S100‐β‐positive RSC96 cells (Supporting Information S1: Figure S7). The migration capacity of RSC96 cells was then investigated in the ADSCs and RSC96 cells co‐culture system. Through continuous monitoring of scratch experiments, the reduction in scratch area was attributed to the collective impact of cell migration and cell proliferation. In order to eliminate the confounding influence of cell proliferation on cell migration analysis, RSC96 cells were exposed to mitomycin at concentrations of 0.15, 0.3, 0.5, 0.75, and 1 μg/mL to inhibit cell division, thereby attributing the observed effects solely to cell migration. Subsequently, cell proliferation was evaluated using the EdU assay; it was shown that in comparison with the mitomycin‐treated group at a concentration of 0.15 μg/mL (9.49 ± 0.99), the groups treated with mitomycin at 0.3 (6.34 ± 0.64), 0.5 (0.87 ± 0.17), 0.75 (0.34 ± 0.14), and 1 μg/mL (0.32 ± 0.17) significantly suppressed the proliferation of RSC96 cells (Supporting Information S1: Figure S6A,B). However, the results of cell counting indicated that excessive mitomycin concentrations could potentially impact cell viability and growth (Supporting Information S1: Figure S6C). Therefore, a concentration of 0.3 μg/mL of mitomycin was chosen for treating RSC96 cells in subsequent scratch experiments to eliminate the influence of cell proliferation on migration efficacy. Afterwards, ADSC and RSC96 cells were co‐cultured using transwell systems to assess the horizontal migration of RSC96 cells. After allowing cells to migrate for 72 h, the wound area was examined. The results from scratch assay revealed a reduction of approximately 59.19% in the wound area of the ADSC and RSC96 cells co‐culture system after 24 h compared to the wound area at 0 h (Figure 3D–H). After 48 h, the wound area in the ADSC group decreased by roughly 77.90% in contrast to the wound area at 0 h. At 72 h, the wound area in the ADSC group was nearly fully closed, whereas the control group retained 38.47% of the wound area. This indicated a significant enhancement in RSC96 cells' migration facilitated by ADSC.

### 3.4 Effects of ADSC/SCNT/RAM on the Differentiation of PC12 Cells Into Neurons

**FIGURE 4:**
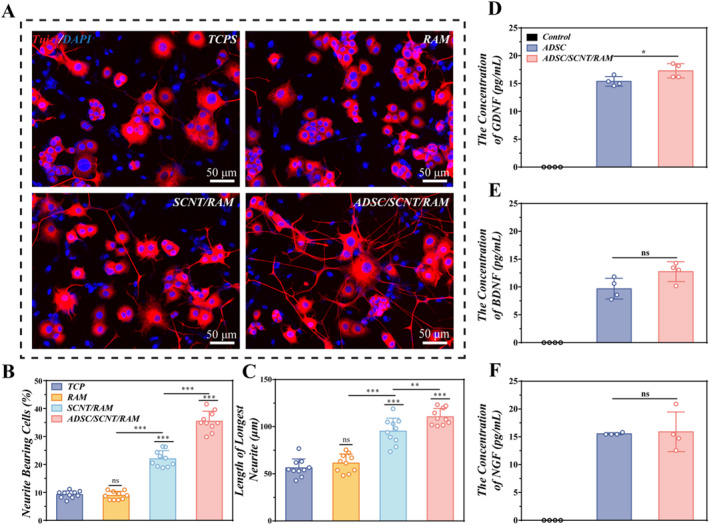
ADSC/SCNT/RAM promotes the differentiation of PC12 cells into neurons. (A) Immunofluorescence staining of PC12 cells after 5 days of differentiation on TCPs, RAM, SCNT/RAM and ADSC/SCNT/RAM. Tuj‐1 (red) labeled neurons and DAPI (blue) labeled nuclei. Scale bar: 50 μm. (B, C) Percentage of cells carrying neurite (B) and length of neuromasts (C) cultured on TCPs, RAM, SCNT/RAM, and ADSC/SCNT/RAM. (D–F) Levels of GDNF (D), BDNF (E), and NGF (F) in the supernatant after 3 d of ADSC culture.

ADSCs are capable of secreting a substantial quantity of neurotrophins during peripheral nerve repair, including NGF, BDNF, and GDNF, all of which are critical for the regeneration of injured neurons. To investigate whether culture on an SCNT/RAM scaffold influences the secretory capacity of ADSCs for neurotrophins, we quantified the levels of GDNF, BDNF, and NGF in the conditioned media. Media samples were collected from three sources: blank culture medium, ADSCs cultured on standard tissue culture plates (TCPs), and ADSCs cultured on the SCNT/RAM scaffold. After 3 days, ELISA analysis revealed that ADSCs on the SCNT/RAM scaffold secreted significantly higher levels of GDNF (17.30 ± 0.64 pg/mL) than those cultured on TCPs (15.40 ± 0.43 pg/mL). In contrast, the secretion levels of BDNF and NGF from ADSC/SCNT/RAM were comparable to those from plate‐cultured cells, with no statistically significant differences observed (BDNF: 12.76 ± 0.89 pg/mL vs. 9.69 ± 0.93 pg/mL) and NGF (15.91 ± 1.78 pg/mL vs. 15.55 ± 0.09 pg/mL). These findings indicate that culture on the SCNT/RAM scaffold does not impair and may even enhance the ability of ADSCs to secrete crucial neurotrophins (Figure 4D–F). To further investigate the impact of ADSC/SCNT/RAM on nerve axon growth, a co‐culture model was established using the Transwell system. PC12 cells were inoculated on various substrates, including the TCP, RAM, SCNT/RAM, and ADSC/SCNT/RAM. After 5 days of culture, neurites were stained with Tuj‐1 immunofluorescence. The fluorescent images revealed that PC12 cells in the TCP and RAM groups were almost difficult to differentiate, whereas PC12 cells in the SCNT/RAM and ADSC/SCNT/RAM groups differentiated distinct neural protrusions (Figure 4A). Subsequently, the cell counts bearing neurites and the length of these neurites were statistically analyzed using ImageJ software. The SCNT/RAM group (22.03 ± 0.92%) and the ADSC/SCNT/RAM group (35.46 ± 1.14%) demonstrated a significantly increased sensitivity to neuromasts compared to the TCP group (9.27 ± 0.40%) and RAM group (8.92 ± 0.47%). Additionally, a greater number of cells bearing neurites were observed, with the highest neuronal differentiation efficiency found in the ADSC/SCNT/RAM group (Figure 4B). In addition, the average length of neurites in the ADSC/SCNT/RAM group was 109.80 ± 2.82 μm, significantly longer than that observed in the TCP group (54.37 ± 1.82 μm), RAM group (59.02 ± 1.93 μm) and SCNT/RAM group (95.70 ± 2.66 μm) (Figure 4C). These results indicated that ADSC/SCNT/RAM effectively stimulated neuronal differentiation and neural synapse growth by secreting neurotrophic factors and transmitting electrical signals. These findings suggest that the NGC prepared with ADSC/SCNT/RAM can serve as an effective platform for repairing sciatic nerve injuries.

### 3.5 ADSC/SCNT/RAM NGCs Promote Sciatic Nerve Regeneration in PNI Rats

**FIGURE 5:**
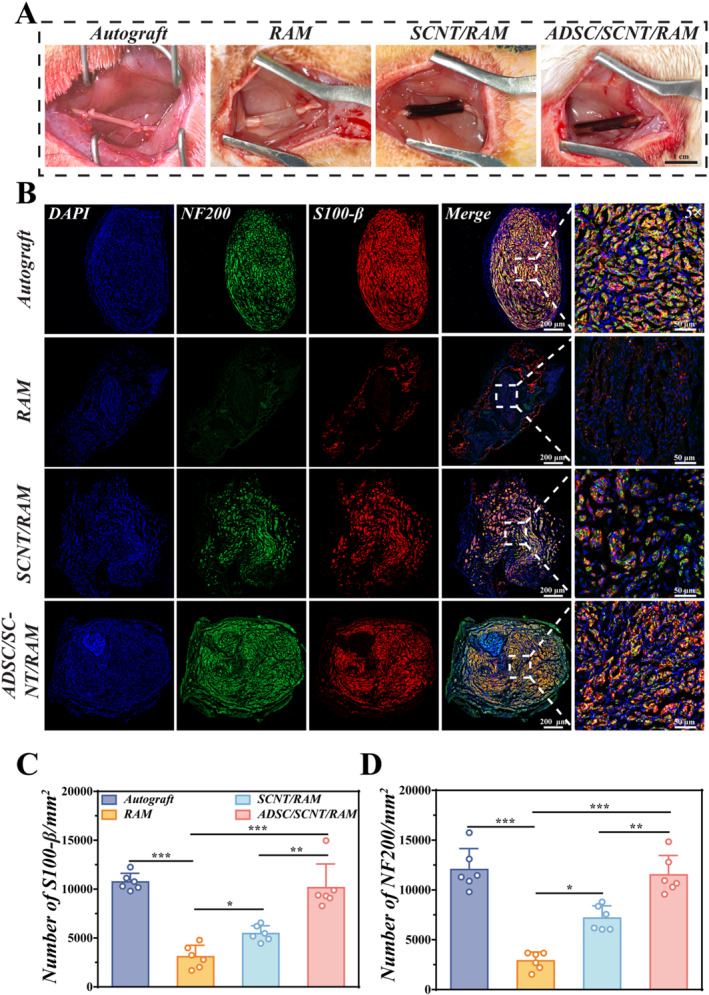
Surgical implantation of NGCs and assessment of sciatic nerve regeneration. (A) Surgical photographs of NGC implantation in the Autograft, RAM, SCNT/RAM, and ADSC/SCNT/RAM groups; Scale bar: 1 cm. (B) Immunofluorescence staining images of NF200 and S100‐β in regenerated sciatic nerve cross‐sections of rats after 12 weeks of repair in the Autograft, RAM, SCNT/RAM, and ADSC/SCNT/RAM groups. Scale bar: 200 μm, 50 μm. (C, D) Quantification of S100‐β‐positive (C) and NF200‐positive (D) cells was performed using immunofluorescence image analysis. *n* = 6.

The regenerative process of peripheral nerves is significantly hindered by the inherent challenges associated with the growth and structural remodeling of damaged axons. Given the exceptional efficacy of ADSC/SCNT/RAM substrates in promoting cell migration and nerve regeneration, further investigation was undertaken to assess the applicability of NGCs based on ADSC/SCNT/RAM for repairing sciatic nerve defects. Initially, we established a 10‐mm sciatic nerve defect model in SD rats, after which the various NGCs were implanted into the nerve defect for 12 weeks. The autograft group served as a positive control (Figure 5A). S100‐β is a marker for neuroglia (specifically SCs), while NF200 is an indicator of myelinated neurons. To evaluate the nerve regeneration regulated by ADSC/SCNT/RAM NGCs at the tissue level, we performed immunofluorescence staining for S100‐β and NF200 on cross‐sections of damaged nerves.

To provide quantitative morphometric evaluation of nerve regeneration—including assessments of axonal density and myelination—we performed quantitative analysis on these images. The immunofluorescence results suggested the expression of S100‐β and NF200 in all animals, indicating successful axon and myelin sheath formation (Figure 5B). Critically, examination of the NF200‐positive axons in the ADSC/SCNT/RAM and Autograft groups revealed a continuous, aligned, and densely bundled morphology that was intimately associated with S100‐β‐positive Schwann cells. This highly organized architecture is indicative of advanced maturation in regenerating nerve fibers. Notably, the ADSC/SCNT/RAM group exhibited closely arranged S100‐β‐positive cells, with a density (10,152 ± 986.00) significantly higher than that of the RAM group (3084 ± 479.90) and the SCNT/RAM group (5465 ± 324.30), while being similar to that of the autograft group (10,737 ± 357.10) (Figure 5C). Immunohistochemical staining and subsequent quantification of NF200 within regenerated nerve cross‐sections yielded results that were comparable to those obtained from S100‐β staining analysis. In particular, the density of NF200‐positive cells in the ADSC/SCNT/RAM group was (11,517 ± 795.70), significantly exceeding that in the RAM group (2881 ± 363.80) and the SCNT/RAM group (7168 ± 505.00), and similar to that in the Autograft group (12,053 ± 857.00) (Figure 5D). These findings clearly demonstrate that ADSC/SCNT/RAM NGC supported the regeneration of damaged sciatic nerve axons.

### 3.6 ADSC/SCNT/RAM NGCs Promote Motor Function Recovery in PNI Rats

**FIGURE 6:**
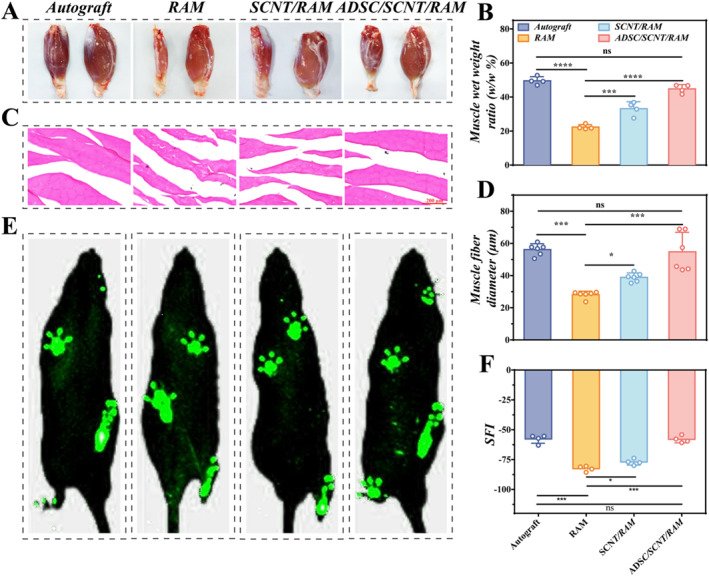
The promotion of motor function recovery in PNI rats following ADSC/SCNT/RAM NGC implantation. (A) Photographs of the left and right gastrocnemius muscles from rats in the Autograft, RAM, SCNT/RAM, and ADSC/SCNT/RAM groups. (B) Wet‐to‐weight ratios of muscles of rats in Autograft, RAM, SCNT/RAM, and ADSC/SCNT/RAM groups. (C) H&E staining images of muscles in the Autograft, RAM, SCNT/RAM, and ADSC/SCNT/RAM groups. (D) Statistical graphs of muscle fiber diameters in the Autograft, RAM, SCNT/RAM, and ADSC/SCNT/RAM groups. (E) Walking footprints of SD rats in the Autograft, RAM, SCNT/RAM, and ADSC/SCNT/RAM groups. (F) SFI of the Autograft, RAM, SCNT/RAM, and ADSC/SCNT/RAM groups.

To evaluate motor function recovery post‐NGC transplantation, the sciatic nerve function index (SFI) was employed subsequently. The SFI provides a quantitative measure, where values of −100 signify complete motor impairment and 0 signify normal function. The footprints and gait of all four groups, the Autograft, RAM, SCNT/RAM, and ADSC/SCNT/RAM groups, were automatically recorded at 12 weeks using a walkway gait analysis system (Figure 6E). Subsequently, motor function recovery in PNI rats was evaluated by calculating the SFI. Gait map analysis showed comparable toe extension between the Autograft and ADSC/SCNT/RAM groups, although the extension was greater in these groups than in the RAM and SCNT/RAM groups. Additionally, the SFI values for the RAM group (−82.54 ± 1.15) and SCNT/RAM group (−77.03 ± 1.27) were inferior to those of the Autograft group (−57.73 ± 1.80) and the ADSC/SCNT/RAM group (−58.06 ± 1.46). Notably, the SFI in the ADSC/SCNT/RAM group corresponded to that of the Autograft group, indicating that the motor functional recovery achieved in animals from the ADSC/SCNT/RAM group reached a level similar to that observed in the Autograft group (Figure 6F).

The processes of sciatic nerve regeneration and motor function recovery are closely associated with and depend on the regeneration of the target muscle tissue. 12 weeks post‐implantation, the right and left gastrocnemius muscles were harvested to assess the degree of muscle regeneration and the wet‐to‐weight ratios of the muscle tissues. Significant muscle atrophy was observed in both the RAM and SCNT/RAM groups. In contrast, the Autograft and ADSC/SCNT/RAM groups showed substantial muscle recovery (Figure 6A). The wet weight ratios for the Autograft group (49.58 ± 1.12%) and the ADSC/SCNT/RAM group (57.73 ± 1.80%) were statistically comparable; both groups had ratios that were higher than those of the RAM group (22.35 ± 0.71%) and the SCNT/RAM group (44.81 ± 1.25%) (Figure 6B). Moreover, the muscle regeneration of rats was also determined by H&E staining. It was suggested that the diameter of the muscle fibers was significantly larger in the Autograft group (56.17 ± 1.49 μm) and the ADSC/SCNT/RAM group (54.79 ± 4.93 μm) compared to the RAM group (28.11 ± 0.90 μm) and the SCNT/RAM group (38.91 ± 1.11 μm) (Figure 6C,D). Collectively, these results suggest that the prepared ADSC/SCNT/RAM NGCs have a potential application as tissue scaffolds in peripheral nerve regeneration. While our ADSC/SCNT/RAM NGCs facilitated significant overall motor recovery, as evidenced by improved Sciatic Functional Index (SFI) scores and paw print area, the restoration of fine motor control, particularly complete toe extension, remained suboptimal. This observation is consistent with the well‐documented challenges in peripheral nerve regeneration, where complete functional restitution is rarely achieved. The incomplete recovery can be attributed to several intrinsic limitations of the repair process, including axonal misdirection during regeneration, irreversible muscle atrophy from prolonged denervation, and the absence of a structured rehabilitation protocol. Therefore, while the significant improvement in gross motor function validates the therapeutic potential of our conduits, the persistent deficit in fine motor control highlights a critical area for future optimization in nerve repair strategies.

## 4 Discussion

In conclusion, we combined SS‐modified SCNT and ADSC with RAM hydrogel film to develop an active NGC for neural regeneration. The NGC exhibited excellent electrical conductivity and mechanical durability, whereas the ADSCs embedded within its inner wall continuously released neurotrophic factors, including GDNF, BDNF, and NGF. In vitro studies demonstrated that the prepared ADSC/SCNT/RAM NGC could continuously secrete neurotrophic factors and effectively promote PC12 cells' differentiation, as well as proliferation and migration of SCs. Further in vivo experiments demonstrated the feasibility of the ADSC/SCNT/RAM NGCs in a 10‐mm sciatic defect model in rats. Gait observation and tissue immunohistochemistry results indicated that the ADSC/SCNT/RAM NGC could effectively promote axonal and myelin regeneration, which in turn promoted muscle recovery and motor function recovery. The histological markers provided compelling structural evidence. Although NF200 alone does not confirm function, the regenerated axons exhibited an aligned, bundled morphology intimately associated with S100‐β‐positive Schwann cells (Figure 5B), a hallmark of maturity. These qualitative findings were confirmed by quantitative analyses, which revealed significant, autograft‐comparable increases in axonal density and myelinating cell activity (Figure 5C,D). The definitive evidence for regeneration, however, comes from the strong concordance between these multifaceted structural improvements and the significant recovery in the sciatic functional index (Figure 6), thereby providing a compelling validation of our approach. Our findings, which demonstrate enhanced axonal regrowth and functional recovery in a critical‐size rat defect model, advance upon existing conductive and cell‐based NGCs by offering a more comprehensive biomimetic solution. While future work must address long‐term cell tracking, ultrastructural analysis (e.g., via TEM), and validation in larger animal models, this conductive, living NGC represents a highly promising and multifaceted strategy poised to bridge the gap in current treatments for severe peripheral nerve injuries.

## Author Contributions

R.J.C., T.T.L., H.W., Y.W., and Y.N.H. conceived the idea and designed the experiment. H.C., Y.N.H., M.H.L., and X.Y.P. conducted experiments and data analysis. H.C., H.Z., M.H.Y., and B.Z. assisted with animal experiment. H.C., Y.N.H., and R.J.C. wrote the manuscript.

## Ethics Statement

The animal experiments were reviewed and approved by the Laboratory Animal Ethics Committee of Southeast University (approval number: 20241225002).

## Conflicts of Interest

The authors declare no conflicts of interest.

## Supporting information


Supporting Information S1

## Data Availability

The datasets generated during the current study are available from the corresponding author on reasonable request.
