# Supplementary material for: Conductive Nerve Guidance Conduits Loaded With Adipose Mesenchymal Stem Cells for Peripheral Nerve Regeneration
Source: Smart Med. 2025 Dec 10;4(4):e70025. doi: 10.1002/smmd.70025 (PMC12697924; doi:10.1002/smmd.70025)
Supplement: Supplementary file 1 — Supporting Information S1 [file SMMD-4-e70025-s001.docx]

Supporting Information

Conductive nerve guidance conduits loaded with adipose mesenchymal stem cells for peripheral nerve regeneration

Hong Cheng^1, 2, #^, Yangnan Hu^1, 3, #, *^, Menghui Liao^1, 3, #^, Xinyi Pang^4, #^, Hui Zhang^1^, Meihan Yu^5^, Bin Zhang^1^, Yu Wang^6, *^, Huan Wang^7, *^, Tingting Liu^1, *^, Renjie Chai^1, 2, 8, 9, 10, *^

^1^Department of Otolaryngology Head and Neck Surgery, Zhongda Hospital, State Key Laboratory of Digital Medical Engineering, Jiangsu Provincial Key Laboratory of Critical Care Medicine, School of Public Health, School of Medicine, Advanced Institute for Life and Health, Southeast University, Nanjing 210096, China.

^2^Co-Innovation Center of Neuroregeneration, Nantong University, Nantong 226001, China.

^3^School of Medical Engineering, Affiliated Zhuhai People’s Hospital, Beijing Institute of Technology, Zhuhai, 519088, China.

^4^College of Food Science and Engineering, Nanjing University of Finance and Economics, Nanjing 210023, China.

^5^Department of Otolaryngology Head and Neck Surgery, Nanjing Drum Tower Hospital, Clinical Medical College of Traditional Chinese and Western Medicine, Nanjing University of Chinese Medicine, Nanjing 210023, China.

^6^Wenzhou Institute, University of Chinese Academy of Sciences, Wenzhou, Zhejiang 325001, China.

^7^The Eighth Affiliated Hospital, Sun Yat-Sen University, Shenzhen, 518033, China.

^8^Department of Neurology, Aerospace Center Hospital, School of Life Science, Beijing Institute of Technology, Beijing 100081, China.

^9^Institute for Stem Cell and Regeneration, Chinese Academy of Science, Beijing, China.

^10^Southeast University Shenzhen Research Institute, Shenzhen 518063, China.

E-mail: Yangnan Hu (yangnanhu@163.com); Yu Wang (yuwang@wiucas.ac.cn); Huan Wang [(wangh679@mail.sysu.edu.cn);](mailto:(wangh679@mail.sysu.edu.cn);) Tingting Liu [(tingtingliu@seu.edu.cn);](mailto:(wangh679@mail.sysu.edu.cn);) Renjie Chai (renjiec@seu.edu.cn)


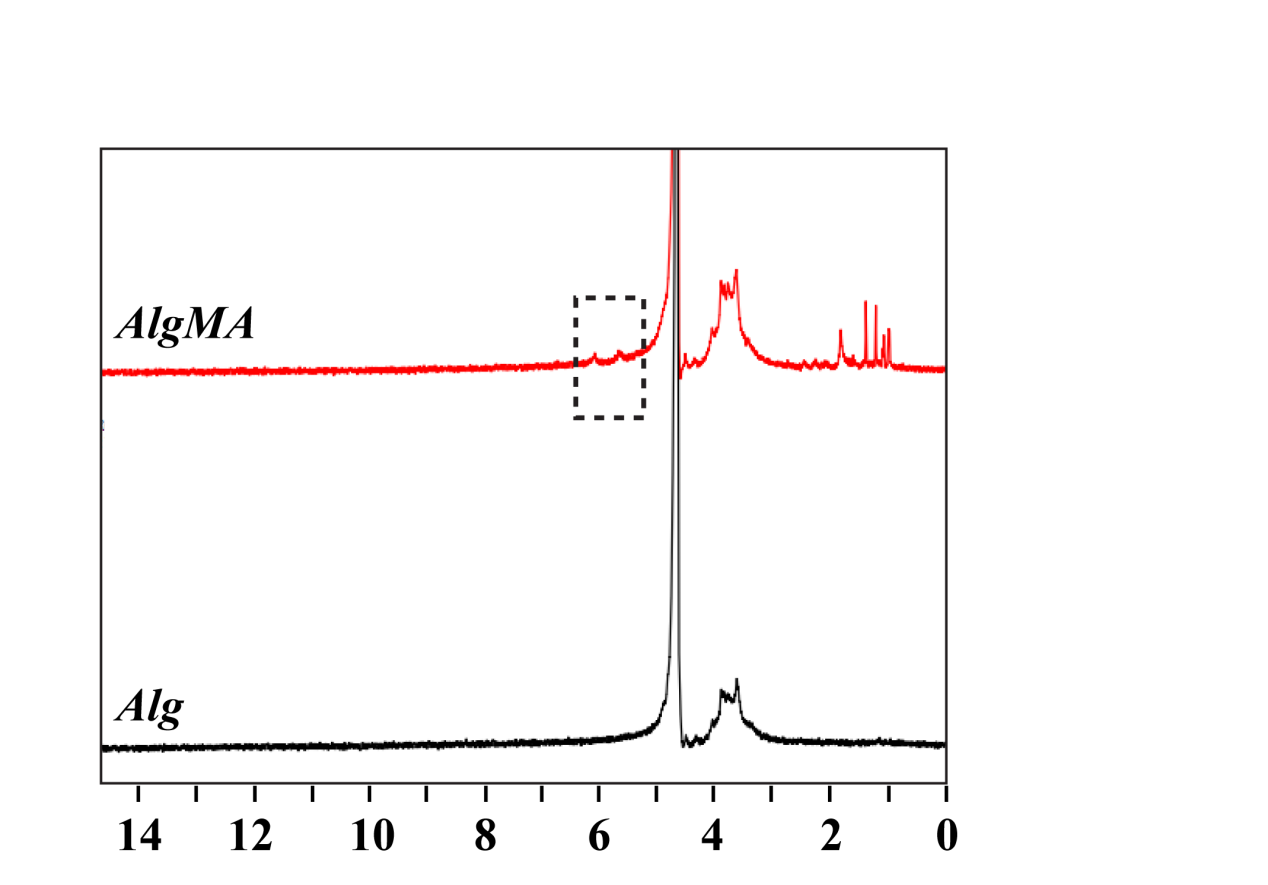


**Figure S1.** Hydrogel ^1^H NMR Hydrographic Analysis.


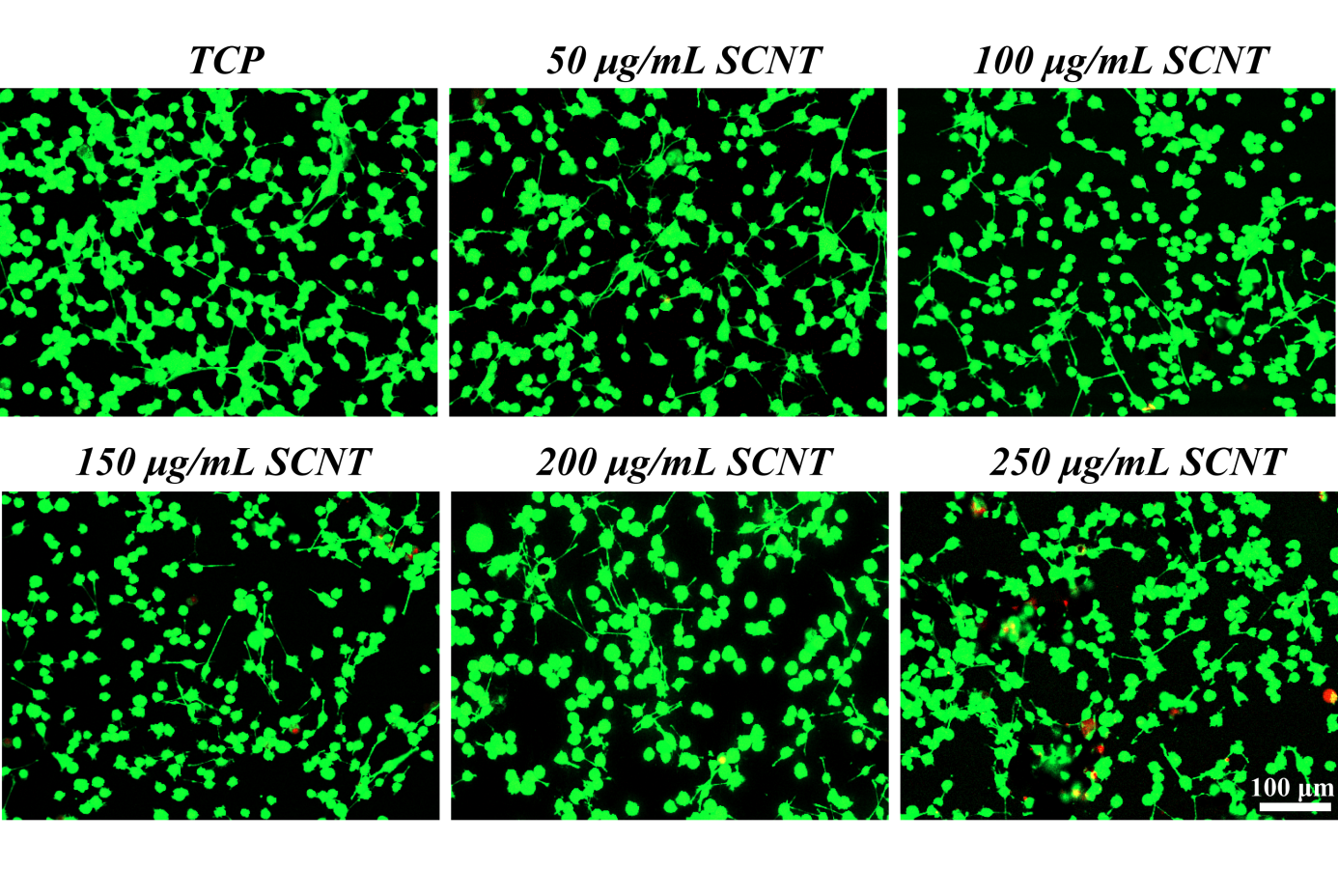


**Figure S2.** Live-dead staining images of RSC96 cells grown on different concentrations of SCNT/RAM, green: live cells; red: dead cells. Scale bar: 100 μm.


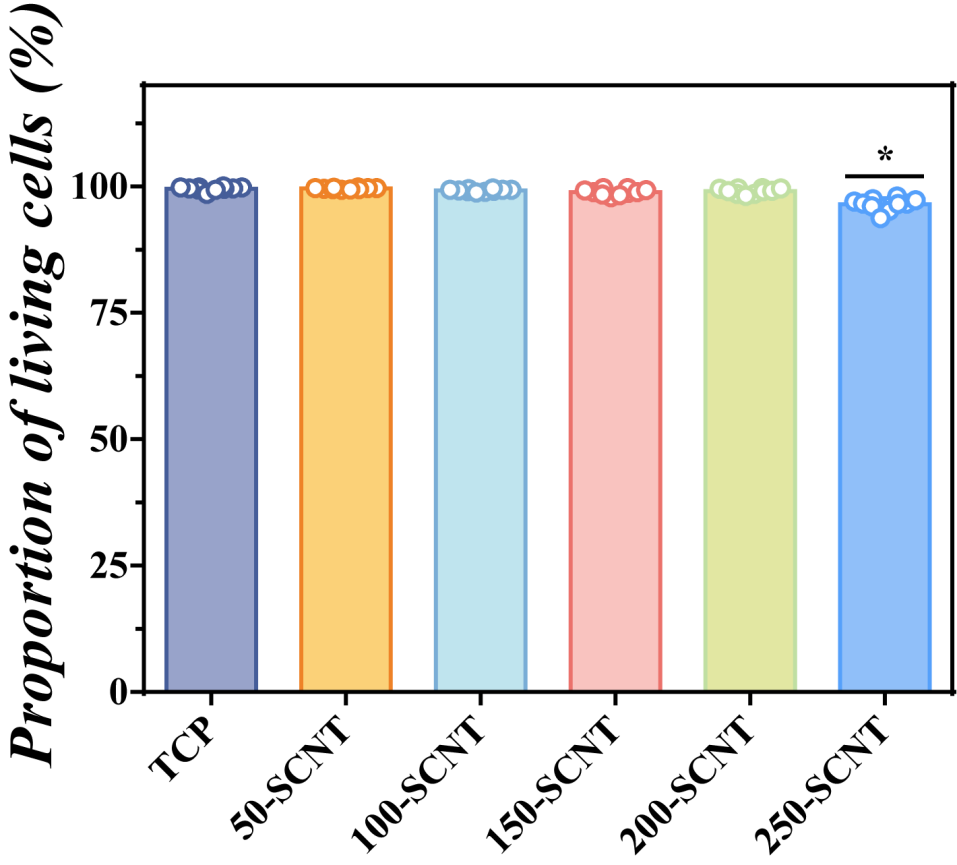


**Figure S3.** RSC96 cells live-dead staining statistics.


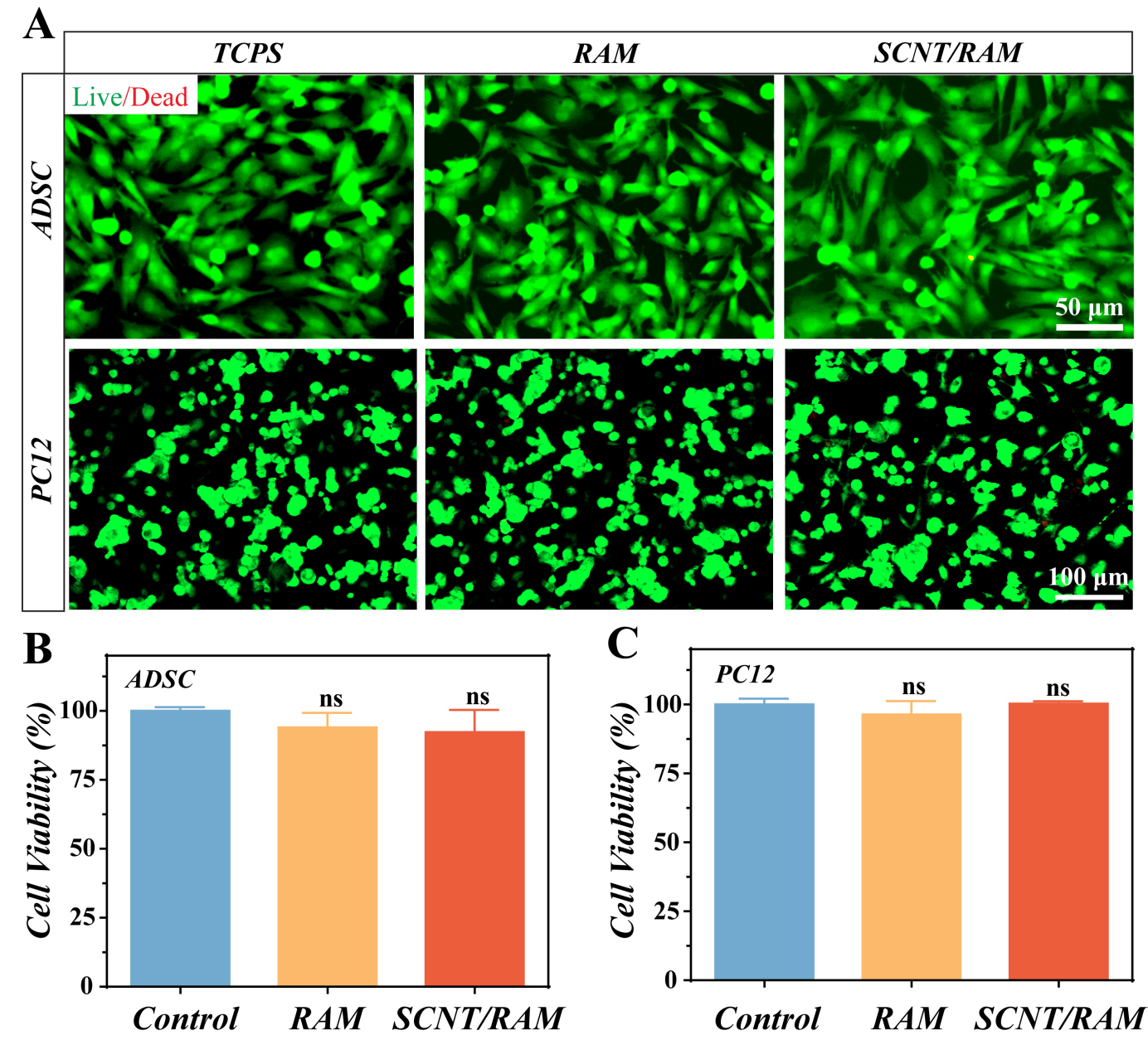


**Figure S4.** Biocompatibility of SCNT/RAM for ADSC and PC12 cells growth. (A) Live-dead staining images of ADSC (Scale bar: 50 μm) and PC12 cells (Scale bar: 100 μm) grown on TCP, RAM and SCNT/RAM, green: live cells; red: dead cells; (B) Cell viability of ADSC cells detected by CCK-8 method; (C) Cell viability of PC12 cells detected by CCK-8 method.


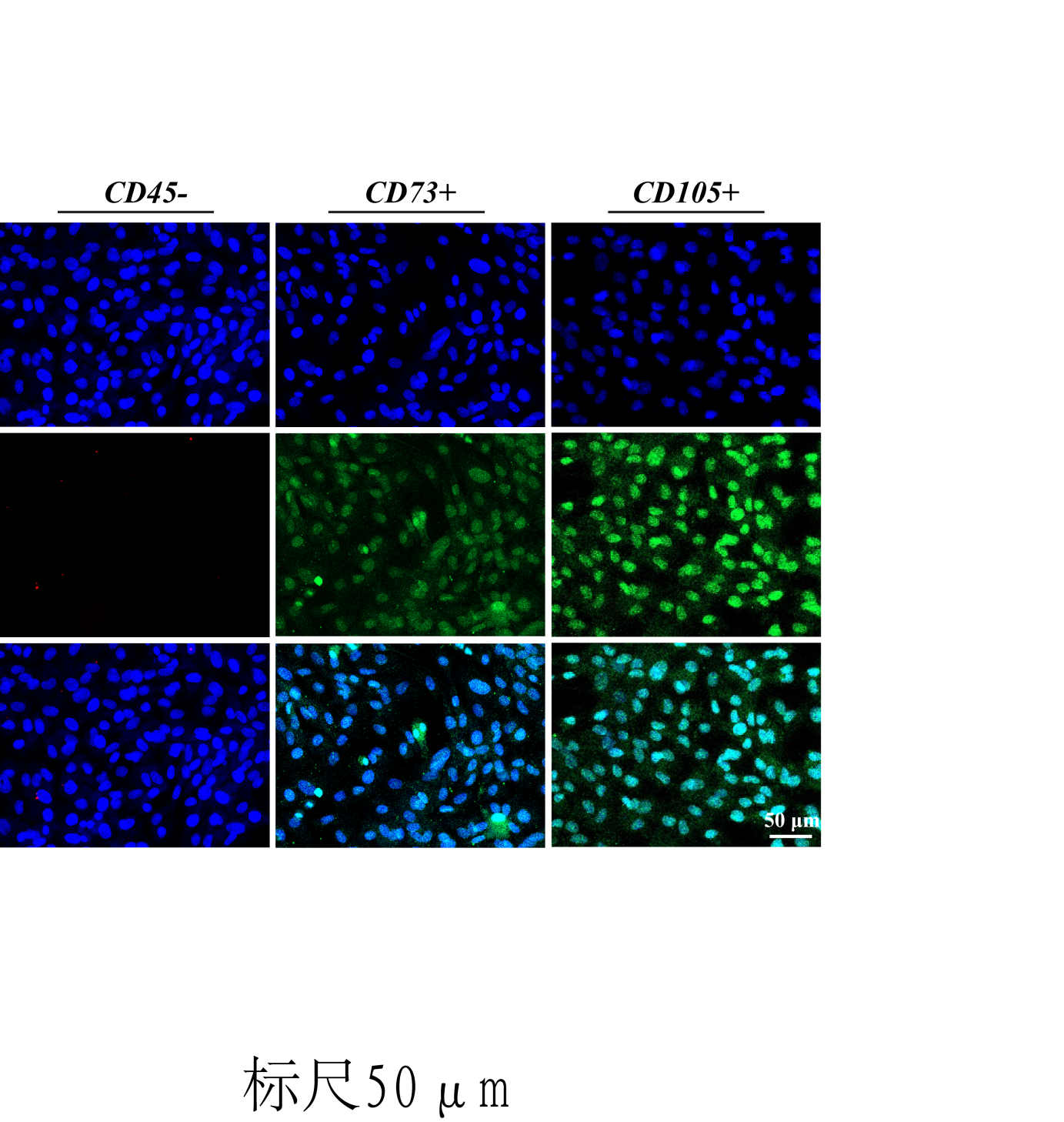


**Figure S5.** Immunofluorescence images of ADSC. Scale bar: 50 μm.


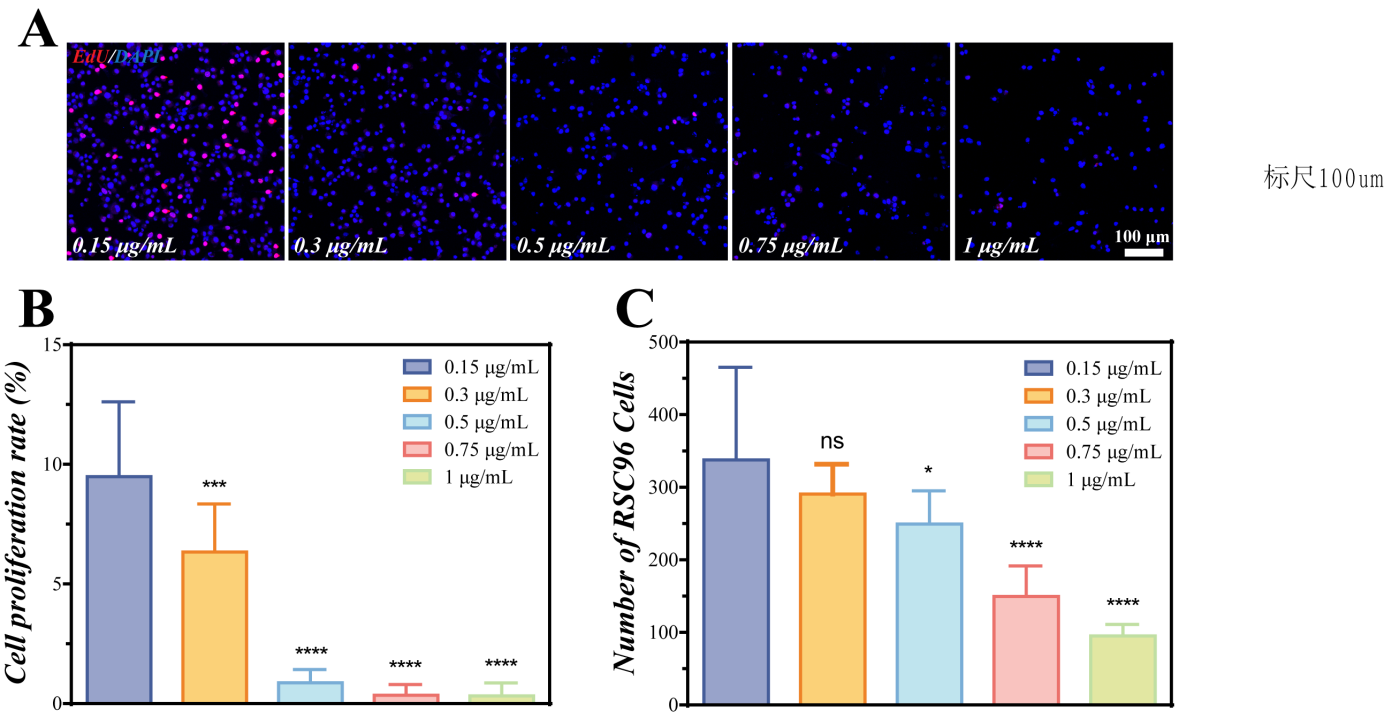


**Figure S6.** Inhibition of RSC96 cells proliferation by mitomycin. (A) Fluorescence images of RSC96 cells proliferation under 0.15, 0.3, 0.5, 0.75, and 1 μg/mL mitomycin treatment. EdU (red) labeled the nuclei of dividing cells, and DAPI (blue) labeled the nuclei of all cells. Scale bar: 100 μm; (B) statistics of RSC96 cells proliferation rate; (C) statistics of RSC96 cells number.


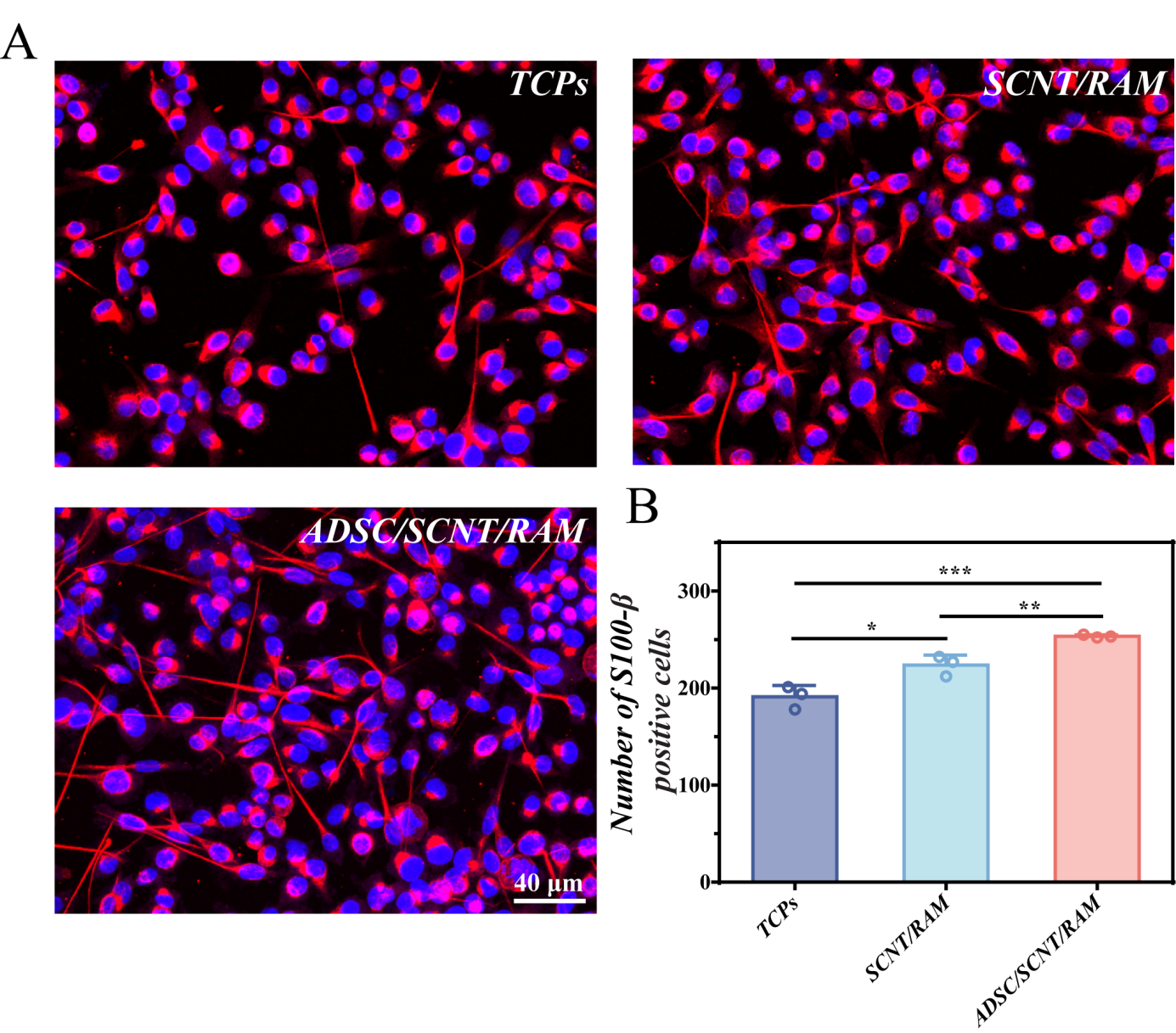


**Figure S7.** ADSC/SCNT/RAM promotes the proliferation of S100-β-positive RSC96 cells. (A) Immunofluorescence images of S100-β-positive RSC96 cells. S100-β (red), DAPI (blue); (B) Cell number of S100-β-positive RSC96 cells.
